# Polymorphisms in *NLRP1* Gene Are Associated with Type 1 Diabetes

**DOI:** 10.1155/2019/7405120

**Published:** 2019-07-14

**Authors:** Xiaoxiao Sun, Ying Xia, Yue Liu, Yanfei Wang, Shuoming Luo, Jian Lin, Gan Huang, Xia Li, Zhiguo Xie, Zhiguang Zhou

**Affiliations:** ^1^Department of Metabolism and Endocrinology, The Second Xiangya Hospital, Central South University, Changsha, Hunan, China; ^2^Key Laboratory of Diabetes Immunology, Central South University, Ministry of Education, National Clinical Research Center for Metabolic Diseases, Changsha, Hunan, China

## Abstract

**Objective:**

The aim of this study was to clarify the association of two single-nucleotide polymorphisms (SNPs) (rs11651270 and rs2670660) in the *NLRP1* (NLR family pyrin domain containing 1) gene with type 1 diabetes (T1D) in the Chinese Han population. We hypothesize that mutations in the *NLRP1* gene may affect the susceptibility to T1D.

**Materials and Methods:**

A case control study was designed, and participants fulfilling the diagnostic criteria of classical T1D as well as nondiabetic controls were enrolled in the study. The polymorphisms rs11651270 and rs2670660 were genotyped by polymerase chain reaction (PCR) and Sanger sequencing. Chi-squared test and logistic regression analysis were performed to compare the distributions of the allele and genotype between cases and controls. Kruskal-Wallis one-way ANOVA was used to compare the characteristics of different genotypes in participants with T1D.

**Results:**

A total of 510 participants with classical T1D as well as 531 nondiabetic controls were enrolled in the study. The two groups were matched in sex (*p* = 0.418). The age (*p* < 0.001) and BMI (*p* < 0.001) were significantly lower in cases compared to controls. Significantly higher values were observed for fasting plasma glucose (FPG) (*p* < 0.001) and 2 h postprandial plasma glucose (PPG) (*p* < 0.001) in individuals with T1D. Regarding the allelic model, the minor allele C of rs11651270 was significantly associated with lower risk of T1D compared with the T allele (OR = 0.714, 95% CI = 0.579-0.882). Both rs11651270 and rs2670660 polymorphisms were associated with T1D in the Chinese Han population under a dominant model (OR = 0.648, 95% CI = 0.503-0.834 and OR = 0.716, 95% CI = 0.549-0.934, respectively) and an overdominant model (OR = 0.663, 95% CI = 0.511-0.860 and OR = 0.711, 95% CI = 0.541-0.935, respectively). Additionally, the polymorphism rs11651270 was also related to T1D in an additive model (OR = 0.719, 95% CI = 0.583-0.887). Most importantly, when we analyzed the clinical characteristics of T1D individuals with different genotypes, we found that the age of onset with the TT genotype at rs11651270 was younger than those with the other two genotypes (*p* = 0.001).

**Conclusions:**

SNPs in the *NLRP1* gene were associated with T1D, as well as the age of onset in the Chinese Han T1D individuals. Our study indicated that the *NLRP1* gene might play a pivotal role in the etiopathogenesis of T1D and could be applied to genetic screening of T1D in the Chinese Han population.

## 1. Introduction

Type 1 diabetes is an organ-specific autoimmune disease characterized by severe autoimmune destruction of insulin-producing pancreatic *β* cells induced by self-attacking T lymphocytes and inflammatory cytokines [1]. Although the pathogenesis of T1D has not been fully elucidated, T1D is currently considered to be a multifactorial disease resulting from interactions between genetic susceptibility and environmental factors [2]. Human leukocyte antigen (*HLA*) regions, such as *DQ* and *DR*, are demonstrated to be the most important genes affecting T1D susceptibility, which contribute to 40-50% of the genetic risk of T1D [3]. Recently, large-scale genome-wide association studies (GWAS) in Europeans have revealed that more than 50 non-*HLA* genetic loci also affected the susceptibility of T1D [4]. The associations of *HLA* or non-*HLA* genes with T1D in Chinese individuals are to some degree different from those in Caucasians. It has been reported that susceptible *HLA* alleles in Caucasians, such as *DR3* and *DR4*, contributed to much lower risk in Chinese individuals, while the *DR9* allele which was not related to T1D in Caucasians conferred a high risk of the disease in Chinese individuals [5].

Innate immunity and inflammation are involved in the pathogenesis and progress of T1D [6]. IL-1*β* and IL-18 are two important proinflammatory cytokines, and the cleavage and production of these cytokines are mediated by inflammasomes [6]. Several members of the nucleotide-binding oligomerization domain-like receptor (NLR) family are major components of inflammasomes which are cytoplasmic complexes sensing pathogen- or danger-associated molecular patterns (PAMPs/DAMPs) and activating caspase-1 [7]. Due to its critical role, inflammasome has been found to be involved in autoimmunity and chronic inflammation [7]. As one of the best characterized inflammasome-forming NLR family members, NLRP1 (NLR family pyrin domain containing 1) inflammasome is considered to be a reasonable and effective target for modulating the initiation and progression of various autoimmune disorders [8].

The *NLRP1* gene is located in chromosome 17p13.2 in humans. High expression of this gene in immune cells indicates its role in the regulation of the immune system [9]. Many studies have shown that *NLRP1* polymorphisms are implicated in several autoimmune diseases, including rheumatoid arthritis, systemic sclerosis, Crohn's disease [10], Addison's disease [11], and vitiligo-associated multiple autoimmune diseases [12]. However, the relationship between *NLRP1* and T1D remains discordant among distinct ethnic groups. For example, a recent study with a large sample in Norway has demonstrated a significant association between a polymorphism in *NLRP1* and T1D [13], while another two studies in Polish and Northeast Brazilian populations did not confirm such an association [14, 15]. Nonetheless, data of *NLRP1* in participants with T1D from Chinese Han population is still lacking. Given that the allelic and/or locus heterogeneity exists among diverse ethnic groups, it is of significance to determine the relationship between SNPs in *NLRP1* and susceptibility to T1D in order to further understand the effect of *NLRP1* on T1D in the Chinese Han population. Two variants in the *NLRP1* gene rs11651270 and rs2670660 were chosen, as rs11651270 is a gain-of-function variant leading to a Met-Val substitution [16] and it has been demonstrated to induce IL-1*β* production [17]. As for polymorphism rs2670660, it is a promoter variation and has been reported to be associated with autoimmune disorders previously [11, 12, 18]. Accordingly, the aim of our study was to evaluate the association between these two specific SNPs in the *NLRP1* gene and T1D in the Chinese Han population. We hypothesize that mutations in the *NLRP1* gene may affect the susceptibility to T1D.

## 2. Materials and Methods

### 2.1. Study Population

This study was approved by the Ethics Committee of the Second Xiangya Hospital of Central South University in accordance with the Declaration of Helsinki guidelines. Written informed consent was obtained from all participants who were fully aware of the study after the goals and procedures were explained.

A total of 510 participants with classical T1D and 531 nondiabetic controls were prospectively recruited into this study, and all of them were of Chinese Han descent. T1D individuals were consecutively recruited from the Department of Metabolism and Endocrinology of the Second Xiangya Hospital at the time of diagnosis. Cases were enrolled in the study if they met the following entry criteria: (1) fulfilling the diagnostic criteria for diabetes developed by WHO in 1999; (2) acute onset and insulin dependency at the time of diagnosis; (3) positive for at least one of the following islet autoantibodies in serum: glutamic acid decarboxylase antibody (GADA), protein tyrosine phosphatase antibody (IA-2A), and zinc transporter 8 antibody (ZnT8A) [19, 20]. Besides, participants with other autoimmune disorders were excluded. The healthy controls were enrolled through epidemiological studies in Hunan province and physical examinations in the Second Xiangya Hospital. Their inclusion criteria were fasting plasma glucose (FPG) < 5.6 mmol/L and 2 h postprandial plasma glucose (PPG) < 7.8 mmol/L in the 75 g oral glucose tolerance test (OGTT). Individuals who had autoimmune diseases, cancer, or family history of diabetes were not included.

### 2.2. Demographic Characteristics and Biochemical Measurements

Anthropometric measurements like weight and height of the individuals were measured by physicians to calculate the body mass index (BMI). Other clinical data including sex, age, and duration of T1D were also collected. HbA1c levels were measured by automated liquid chromatography (HLC-723G8, Tosoh, Japan). The levels of C-peptide were measured by a chemiluminescence method (ADVIA Centaur XP Immunoassay System, Siemens, Germany). Diabetes-associated autoantibodies were measured by a highly sensitive and specific quantitative radioligand binding assay.

### 2.3. DNA Extraction

Peripheral venous blood samples were collected from each participant and kept at -80°C in tubes containing ethylenediaminetetraacetic acid. Genomic DNA of all samples for genotyping was extracted using Genenode whole-blood genomic DNA extraction kit (Genenode Biotech Co. Ltd., Beijing) according to the manufacturer's instructions. The DNA samples were stored at -80°C after extraction.

### 2.4. SNPs Selection and Genotyping

Two SNPs in the *NLRP1* gene (rs11651270 and rs2670660) were selected based on functional effect, minor allele frequency (MAF), and/or previously reported associations with autoimmune disorders.

Genotyping was performed using PCR and Sanger sequencing. Database of SNP (dbSNP) was adopted to find the sequences of the two SNPs. The PCR primers were designed using Primer3 according to the general primer design principles. The detailed sequences were listed in Table 1.

The PCR cycling program was as follows: 94°C for 5 min; 10 cycles of denaturation at 94°C for 30 s, annealing at 62°C with 0.2°C decrements per cycle for 30 s, and extension at 72°C for 45 s; 30 cycles of 94°C for 30 s, 60°C for 30 s, and 72°C for 30 s; and a final extension at 72°C for 5 min, followed by holding at 4°C. The amplified PCR products were performed by Sanger sequencing to identify the genotype of each individual.

### 2.5. Statistical Analysis

The statistical analyses were operated by SPSS version 20.0 for Windows (SPSS Inc., Chicago, IL, USA). Continuous variables were presented as median (interquartile range (IQR)) due to the nonnormality of data, and Mann-Whitney *U* test was used to compare the differences between cases and controls. The differences of categorical data were tested by chi-squared test. The frequencies of the genotype and allele were assessed by direct counting. The distributions of the genotype for the controls were tested for Hardy–Weinberg equilibrium (HWE) using chi-squared goodness-of-fit test. Four genetic models were adopted based on the genotype frequencies of each locus to further evaluate the differences in distributions: dominant, recessive, overdominant, and additive models. The genotype frequencies were statistically compared between the cases and controls for each genetic model by logistic regression analysis, and allele frequencies were estimated using chi-squared test. The association between polymorphisms in the *NLRP1* gene and the risk of T1D was evaluated via odds ratio (OR) and 95% confidence interval (CI). Kruskal-Wallis one-way ANOVA was performed to compare the clinical characteristics of different genotypes in T1D individuals. All *p* values were two tailed, and the statistical significance was defined as *p* < 0.05 for a single test. A formal Bonferroni correction was used for multiple comparisons and *p* value after correction (pc) < 0.05 was considered as statistically significant. The number of multiple comparisons that were corrected in the study was two.

## 3. Results

### 3.1. Clinical and Biochemical Characteristics of Cases and Controls

A total of 1041 participants were selected, including 510 participants with classical T1D and 531 nondiabetic controls. The main clinical and biochemical characteristics were summarized in Table 2. The two groups were matched in sex (male/female) (275/235 vs. 273/258, *p* = 0.418). The analyses on other anthropometric and clinical data revealed that T1D participants enrolled were younger (*p* < 0.001) and leaner (*p* < 0.001) than healthy controls. Significant higher values were observed for fasting plasma glucose (FPG) (*p* < 0.001) and 2 h postprandial plasma glucose (PPG) (*p* < 0.001) in individuals with T1D.

### 3.2. The Allele and Genotype Distributions of *NLRP1* Polymorphisms in Cases and Controls

The results of genotype and allele distributions of rs11651270 and rs2670660 SNPs in *NLRP1* between cases and controls were displayed in Table 3. The distributions of genotype in controls were consistent with HWE for both SNPs.

Lower frequencies of rs11651270 CC and CT genotypes were discovered in cases than those in controls (CC: 4.51% vs. 5.65%, *p* = 0.174, CT: 28.43% vs. 37.48%, *p* = 0.001, respectively). The C allele was present in 18.73% of the case group and 24.38% of the control group. These results showed that the C allele and C-carrier state of rs11651270 seemed to be protective against the development of T1D (OR = 0.714, 95% CI = 0.579-0.882 and OR = 0.643, 95% CI = 0.494-0.838, respectively). All results demonstrated that rs11651270 was correlated with T1D susceptibility and C allele seemed to play a protective role compared with T allele.

For rs2670660, the frequencies of GG, GA, and AA genotypes were 2.75%, 24.12%, and 73.14% in cases, respectively, and were 3.01%, 30.89%, and 66.10% in controls, respectively. The allele frequency of G was present in 14.80% of cases and 18.46% of controls. After Bonferroni correction for multiple comparisons, we found a significant negative association between GA heterozygous and T1D (OR = 0.706, 95% CI = 0.536-0.930) at rs2670660. Besides, a weak association was identified that G allele was related to T1D (OR = 0.768, 95% CI = 0.609-0.968) when the *p* value was not corrected for multiple comparisons. However, this association lost significance after Bonferroni correction.

### 3.3. Association Analysis of *NLRP1* Polymorphisms in Cases and Controls

In order to confirm the role of the two polymorphisms in T1D predisposition, genetic model analyses were performed (Table 4). The results revealed that the presence of the CT genotype for rs11651270 showed a significantly lower risk towards T1D susceptibility (OR = 0.663, 95% CI = 0.511-0.860) under an overdominant model (CT vs. CC+TT), as well as the GA genotype for rs2670660 (OR = 0.711, 95% CI = 0.541-0.935). Besides, when comparing genotype distributions using a dominant model (CC+CT vs. TT and GG+GA vs. AA, respectively), both rs11651270 and rs2670660 showed associations with T1D (OR = 0.648, 95% CI = 0.503-0.834 and OR = 0.716, 95% CI = 0.549-0.934, respectively). Furthermore, in an additive model (CC vs. CT vs. TT), the genotype distributions of rs11651270 significantly differed between cases and controls (OR = 0.719, 95% CI = 0.583-0.887). However, there was no significant association at rs2670660 in an additive model (GG vs. GA vs. AA) after Bonferroni correction for multiple comparisons. As for a recessive model (CC vs. CT+TT, GG vs. GA+AA, respectively), we did not find any associations at both rs11651270 and rs2670660.

### 3.4. Clinical Characteristics of Participants with T1D in Different Genotypes of SNPs in the *NLRP1* Gene

Clinical characteristics in participants with T1D were compared among different genotypes of polymorphisms in the *NLRP1* gene in an exploratory analysis (shown in Table 5). Sex, T1D duration, and body mass index (BMI) did not vary considerably among genotypes of rs11651270 and rs2670660, as well as biochemical characteristics such as fasting C-peptide (FCP), 2 h postprandial C-peptide (PCP), and HbA1c. Besides, the positive rate of three diabetes-associated autoantibodies among different genotypes also showed no significance in both polymorphisms. However, the age of onset was younger in participants with T1D who were homozygous for the rs11651270 TT genotype compared with C allele carriers (*p* = 0.001).

## 4. Discussion

Through this case control study, we investigated the possible association between two candidate SNPs of *NLRP1* and T1D in the Chinese Han population. A significant association at rs11651270 and a suggestive association at rs2670660 were identified in the *NLRP1* gene, indicating that *NLRP1* might play a role in T1D.

T1D is one of the classic examples of organ-specific autoimmune diseases, and its incidence in children has been increasing worldwide [21, 22]. Despite that China remains one of the countries with the lower incidence of T1D globally, the incidence has risen from 0.51 to 1.93 per 100000 persons a year for people within the age of 14 in China [23]. Genetic factors have shown to play a crucial role in the pathogenesis of T1D, and numerous genetic variants related to immunity are included [24–26]. Given the key role of the NLR family in innate immune regulation, the association between SNPs in *NLRP1* and T1D was explored in this study. NLRP1 is a member of the NLR family, and it could form an inflammatory complex called inflammasome, which could activate caspase-5 and caspase-1 that subsequently lead to the formation of the potent proinflammatory cytokines IL-1*β* and IL-18. Genetic variants in *NLRP1* have been reported to correlate to various autoimmune diseases [8]. As most epidemiology studies about T1D reported that the peak incidence of the disease appeared close to puberty [23], individuals that we chose in the control group were much older, which means that they have lower possibility to develop T1D.

To our knowledge, this is the first study investigating the association between rs11651270 in *NLRP1* and susceptibility to T1D. At the same time, we also compare the clinical characteristics of participants with T1D in different genotypes. We detected and recorded many clinical characteristics of T1D individuals, including fasting C-peptide (FCP), 2 h postprandial C-peptide (PCP), the positive rate of islet autoantibodies (GADA, IA-2A, and ZnT8A), GADA titer, and IA-2A titer. The reason why we choose FCP and PCP is that they could reflect the function of islet *β* cells. As for the comparison of islet autoantibodies, they are markers of immune destruction of islet *β* cells and the production and distribution of islet autoantibodies display genetic susceptibility to some degree [27].

Our results showed that the C allele of rs11651270 might play a protective role in the development of T1D. Correspondingly, we found that the age of onset of cases with the TT genotype was younger than those with CC and CT genotypes. This finding suggests that rs11651270 may play an important role in the pathogenesis of T1D. The polymorphism rs11651270 is a missense variation, and it has been demonstrated to induce IL-1*β* production [17], which might thus affect the function of NLRP1 and contribute to the pathogenesis of T1D. Besides, this SNP has been reported to correlated to susceptibility and outcomes of various diseases [28, 29]. It has been demonstrated that rs11651270 was associated with asthma severity in Brazilian children [30] as well as the development of a diabetic kidney in Brazilian T1D individuals [16]. The rs2670660 is a promoter variation, and it may contribute to clinical characteristics of autoimmunity and autoinflammatory phenotypes by an allele-specific gene expression signature [31, 32].

The results reported in our study are not quite consistent with those in studies in other populations and other autoimmune disorders. A study containing 1086 T1D cases and 3273 controls in Norway has demonstrated a significant association between the *NLRP1* gene and T1D [13], while another two studies conducted in Poland and Northeast Brazil did not find such association [14, 15]. Our results showed that rs11651270 was significantly associated with T1D while another study confirmed that it was not related to psoriasis vulgaris [33]. Although we did not find a significant association between rs2670660 and T1D, this SNP has been reported to associate with many other autoimmune disorders, such as generalized vitiligo [18], systemic sclerosis [34], and systemic lupus erythematosus [35]. These differences in results would be useful in understanding how the results of our study contributed to the autoimmune disease literature in the context of previous studies.

The discrepancy among various ethnic origins might be related to the heterogeneity of this polygenetic disease. Furthermore, gene-environment interactions may alter the contribution of T1D susceptibility loci [36]. As for the differences with other autoimmune diseases, the expression of *NLRP1* varies widely in distinct tissues and the effect of SNP could be cell specific. Moreover, recent findings have shown that *NLRP1* could protect against metabolic syndrome [37] as well as colitis-associated tumorigenesis [38], which indicates that NLRP1 possibly acts as a homeostatic factor.

Extending this study to identify significant variants at the genome-wide level is of utmost importance to find genetic factors related to T1D and understand the pathophysiology of it. Moreover, the identification of genetic variants associated with T1D exhibits the potential to allow for tailored prevention and treatment for genetically at-risk individuals of T1D.

There are limitations to our study. Two candidate SNPs of *NLRP1* were performed in this study, which were not able to cover the entire gene. In future studies, we should consider more SNPs and make a more detailed analysis so as to provide enough value for the understanding of T1D. Besides, we excluded individuals who had any other autoimmune diseases, which would be a potential limitation since T1D could occur alongside other autoimmune disorders. Additionally, our study evaluated the association between two SNPs and the presence of T1D rather than elucidated a possible mechanism for this association. Therefore, further studies could focus on the functional evaluations of SNPs to dissect their contribution to T1D. Moreover, our study population was limited to Chinese Han population at a single study site and the results were not able to be representative for other human ethnics.

## 5. Conclusions

In summary, we evaluated the genetic contribution of *NLRP1* to the risk of T1D and the present data revealed a protecting association of the C allele in *NLRP1* gain-of-function variant rs11651270 with T1D. Overall, these findings supported the role of innate immunity in the development of T1D and indicated that *NLRP1* polymorphisms may serve as a useful genetic marker. However, further investigations are needed to fully elucidate whether and how *NLRP1* polymorphisms could affect the expression and function of the *NLRP1* gene in order to explore its role in the pathogenesis of T1D.

## Figures and Tables

**Table 1:** Primer sequences of rs11651270 and rs2670660 in *NLRP1.*

SNP	Primer sequence	Product size (bp)
rs11651270		
Forward	5′-TGGGATCAGGTAGAGGTGGA-3′	389
Reverse	5′-AGAATCGCTTGAACCCAGGA-3′	
rs2670660		
Forward	5′-ATACCCAGGTGTTCAGGAGC-3′	328
Reverse	5′-GCCTGTGTTGTACCTTCAGC-3′	

SNP: single-nucleotide polymorphisms.

**Table 2:** Clinical and biochemical characteristics of cases and controls.

Characteristic	Cases	Controls	*p* value
Sample size	510	531	—
Sex (men/women)	275/235	273/258	0.418
Age (year)	22 (13-34)	42 (32-51)	<0.001^∗^
BMI (kg/m^2^)	18.70 (16.49-20.70)	22.60 (20.40-24.70)	<0.001^∗^
T1D duration (month)	5.00 (0.50-21.50)	—	—
FPG (mmol/L)	8.91 (6.11-14.40)	4.87 (4.45-5.30)	<0.001^∗^
PPG (mmol/L)	14.90 (9.90-20.70)	5.60 (4.70-6.40)	<0.001^∗^
FCP (pmol/L)	77.50 (17.79-164.10)	—	—
PCP (pmol/L)	142.21 (39.60-279.20)	—	—
HbA1c (%)	10.00 (7.80-12.80)	—	—

BMI: body mass index; FPG: fasting plasma glucose; PPG: 2 h postprandial plasma glucose; FCP: fasting C-peptide; PCP: 2 h postprandial C-peptide; HbA1c: glycated hemoglobin.

**Table 3:** Frequencies of the genotype and allele of *NLRP1* polymorphisms between cases and controls in the Chinese Han population.

SNP	Cases (*N* = 510) *n* (%)	Controls (*N* = 531) *n* (%)	OR (95% CI)	*p*	pc (*n* = 2)
rs11651270					
*Genotype*					
CC	23 (4.51)	30 (5.65)	0.677 (0.385-1.191)	0.174	0.348
CT	145 (28.43)	199 (37.48)	0.643 (0.494-0.838)	0.001^∗^	0.002^∗^
TT	342 (67.06)	302 (56.87)	—	—	—
*Allele*					
C	191 (18.73)	259 (24.39)	0.714 (0.579-0.882)	0.002^∗^	0.004^∗^
T	829 (81.27)	803 (75.61)	—	—	—
rs2670660					
*Genotype*					
GG	14 (2.75)	16 (3.01)	0.823 (0.396-1.712)	0.602	—
GA	123 (24.12)	164 (30.89)	0.706 (0.536-0.930)	0.013^∗^	0.026^∗^
AA	373 (73.14)	351 (66.10)	—	—	—
*Allele*					
G	151 (14.80)	196 (18.46)	0.768 (0.609-0.968)	0.025^∗^	0.050
A	869 (85.20)	866 (81.54)	—	—	—

SNP: single-nucleotide polymorphisms; OR: odds ratio; 95% CI: 95% confidence intervals; pc: *p* value after correction for multiple comparisons.

**Table 4:** Models proposed for *NLRP1* polymorphisms between cases and controls in the Chinese Han population.

SNP	Minor allele	Genetic model											
		Dominant model			Recessive model			Overdominant model			Additive model		
		OR (95% CI)	*p*	pc	OR (95% CI)	*p*	pc	OR (95% CI)	*p*	pc	OR (95% CI)	*p*	pc
rs11651270	C	0.648 (0.503-0.834)	0.001^∗^	0.002^∗^	0.789 (0.452-1.377)	0.403	0.806	0.663 (0.511-0.860)	0.002^∗^	0.004^∗^	0.719 (0.583-0.887)	0.002^∗^	0.004^∗^
rs2670660	G	0.716 (0.549-0.934)	0.014^∗^	0.028^∗^	0.909 (0.439-1.881)	0.796	—	0.711 (0.541-0.935)	0.015^∗^	0.030^∗^	0.769 (0.610-0.970)	0.026^∗^	0.052

SNP: single-nucleotide polymorphisms; OR: odds ratio; 95% CI: 95% confidence intervals; pc: *p* value after correction for multiple comparisons; dominant model is Mm+mm vs. MM; recessive model is mm vs. MM+Mm; overdominant model is Mm vs. MM+mm; additive model is MM vs. Mm vs. mm; MM is homozygous dominant; Mm is heterozygous; mm is homozygous recessive.

**Table 5:** Clinical characteristics of participants with T1D in different genotypes of SNPs in the *NLRP1* gene.

Characteristics	SNP							
	rs11651270				rs2670660			
	CC	CT	TT	*p* value	GG	GA	AA	*p* value
Sample size	23	145	342	—	14	123	373	—
Sex (men/women)	12/11	80/65	183/159	0.931	8/6	70/53	197/176	0.710
Age of onset (years)	29 (13-37)	23 (14-33)	17 (10-32)	0.001^∗^	27 (15-35)	21 (13-35)	18 (10-32)	0.057
T1D duration (months)	4.00 (0.23-24.00)	3.00 (0.33-18.00)	5.00 (0.56-24.00)	0.590	0.59 (0.12-6.00)	5.00 (0.47-36.00)	5.00 (0.50-16.00)	0.068
BMI (kg/m^2^)	19.63 (17.94-21.23)	18.80 (16.80-20.80)	18.51 (16.30-20.70)	0.155	19.50 (18.52-20.59)	18.91 (16.80-21.08)	18.60 (16.39-20.67)	0.187
FCP (pmol/L)	93.64 (4.55-169.75)	79.63 (21.18-190.98)	74.00 (16.10-156.07)	0.602	148.60 (53.99-213.88)	79.00 (18.89-164.80)	73.54 (15.92-158.87)	0.184
PCP (pmol/L)	169.83 (15.52-259.69)	152.81 (47.80-318.66)	135.00 (34.90-263.40)	0.402	220.88 (138.68-369.63)	161.30 (40.83-284.34)	133.85 (35.94-261.85)	0.129
HbA1c (%)	10.10 (8.65-14.00)	9.75 (7.73-12.48)	10.10 (7.70-12.80)	0.611	12.50 (9.23-14.35)	9.70 (7.70-12.50)	10.10 (7.70-12.80)	0.153
GADA positive%	91.30%	90.34%	87.39%	0.587	92.86%	86.18%	88.98%	0.611
IA-2A positive%	52.38%	42.42%	48.41%	0.449	38.46%	45.05%	47.81%	0.726
ZnT8A positive%	45.00%	25.62%	32.12%	0.162	35.71%	25.25%	32.46%	0.372
GADA titer (U/mL)	448.33 (248.47-850.88)	328.43 (91.06-788.30)	288.81 (78.79-755.57)	0.289	756.39 (271.01-929.77)	266.86 (78.81-728.13)	313.80 (82.87-765.60)	0.127
IA-2A titer (U/mL)	361.12 (62.69-790.02)	190.66 (45.07-659.52)	186.51 (52.12-663.96)	0.618	62.69 (28.90-575.57)	113.51 (41.42-575.28)	231.99 (59.24-695.60)	0.143

SNP: single-nucleotide polymorphisms; BMI: body mass index; FCP: fasting C-peptide; PCP: 2 h postprandial C-peptide; HbA1c: glycated hemoglobin; GADA: glutamic acid decarboxylase antibody; IA-2A: protein tyrosine phosphatase antibody; ZnT8A: zinc transporter 8 antibody.

## Data Availability

The data used to support the findings of this study are available from the corresponding author upon request.
